# The prevalence of general and abdominal obesity according to sasang constitution in Korea

**DOI:** 10.1186/1472-6882-14-298

**Published:** 2014-08-13

**Authors:** Younghwa Baek, Kihyun Park, Siwoo Lee, Eunsu Jang

**Affiliations:** Department of KM Health Technology Research Group, Korea Institute of Oriental Medicine, 1672 Yuseongdae-ro, Yuseong-gu, Daejeon, 305-811 South Korea

**Keywords:** General obesity, Abdominal obesity, Sasang constitution, Prevalence, Body mass index, Waist circumference, Waist-to-hip ratio

## Abstract

**Background:**

Obesity is an important risk factor for cardiovascular and metabolic diseases and could affect mortality rates. Body mass index (BMI) and waist circumference (WC) have been used to classify obesity, and waist-to-hip ratio (WHR) has recently emerged as a discriminator of cardiovascular disease. Sasang constitution (SC) is a kind of well-known traditional Korean medicine: Tae-eumin (TE), Soeumin (SE), Taeyangin (TY) and Soyangin (SY) carrying a different level of susceptibility to chronic diseases. We aimed to examine the prevalence in general and abdominal obesity (AO) using BMI, WC and WHR according to SC in the Korean population.

**Methods:**

A total of 3,348 subjects were recruited from 24 Korean medicine clinics. Obesity was divided into three categories: general obesity by BMI, abdominal obesity by waist circumference (WC AO) and abdominal obesity by waist-to-hip ratio (WHR AO). A Chi-square test was performed to compare prevalence, and logistic regression was conducted to generate odds ratios (ORs) according to SC (*p* < .05).

**Results:**

The prevalence of general obesity was significantly higher in males than in females. The highest prevalence of general obesity, WC AO and WHR AO were all observed in the TE type, and the SY and SE types were followed in order, for both males and females respectively.

The TE type was highly associated with increased risk of general obesity (OR = 20.2, 95% CI: 12.4-32.9 in males and OR = 14.3, 95% CI: 10.1-20.2 in females), of WC AO (OR = 10.7, 95% CI: 7.2-15.9 in males and OR = 7.5, 95% CI: 5.8-9.6 in females), and of WHR AO (OR = 4.6, 95% CI: 3.3-6.4 in males and OR = 3.8, 95% CI: 2.9-4.9 in females) compared with the SE type. In addition, after controlling for age, social status and eating habits, the ORs were similar to the crude model according to gender and SC.

**Conclusions:**

This study shows that the prevalence of obesity varies according to SC in the Korean population. In particular, the TE type was highly associated with increased ORs for general obesity, WC AO and WHR AO in both genders.

## Background

Obesity increases the likelihood of various diseases, particularly heart disease, type 2 diabetes, obstructive sleep apnea, certain types of cancers and osteoarthritis [1, 2]. Many epidemiological studies provide evidence that general and abdominal obesity increase the prevalence of these diseases [3–5]. Therefore, the World Health Organization (WHO) declared obesity to be one of the most serious public health problems of the 21st century [6].

Obesity is classified as overweight, general obesity, abdominal obesity (AO), visceral fat obesity and other types to estimate body fatness, which in turn is defined by body mass index (BMI), waist circumference (WC), waist-to-hip ratio (WHR), skin fold thickness and bio-impedance [7]. Among them, BMI and WC are the main criteria that have been employed to classify obesity and WHR has recently been suggested to be more significantly associated with the risk of cardiovascular events than WC [8]. These three parameters are strongly associated with diseases and are evaluated as risk factors [9].

The cause of obesity is well known to be a combination of environmental factors, including excessive food energy intake, lack of physical activity and genetic susceptibility [10, 11]. Recently, a study revealed that inherence causes a 1.67-fold increase in the odds of obesity [12]. Genetic susceptibility is deeply associated with the theory of Sasang constitution (SC) in Korea, which is thought to be unchangeable regardless of longevity and life preservation in oriental medicine [13]. Sasang constitutional medicine (SCM) is a tailored traditional Korean system that classifies human beings into the following four constitutions: Tae-eumin (TE), Soeumin (SE), Taeyangin (TY) and Soyangin (SY) [13].

According to SCM, the requisite energy, or the preservative energy related to the most hypoactive viscera or the weakness of each SC type, is considered to be the essential energy necessary to maintain homeostasis. The clearing Yin energy, the warming Yang energy, the dispersive energy and the accumulative energy are the requisite energies for SC types, respectively. Diseases and illnesses are caused by the aggravation of hypoactive visceral functions [14].

Each constitution has different body shape, psychological and physiological characteristics [15, 16]. Therefore, several studies have shown that each constitution is associated with a different susceptibility to chronic diseases, including hypertension, diabetes mellitus and metabolic syndrome; this has been shown not only in cross-sectional studies but also in cohort designs [16–20].

Recently, an experimental study revealed that the TE type is more susceptible to obesity than are the other types, from a genetic perspective [21, 22]. Several clinical studies have also suggested that the TE type is associated with overweight or obesity by merely describing different BMI in the general characteristics according to SC [16–19, 23], which indicates that weight characterizes specific SC. However, these studies did not report the prevalence of obesity as indicated by BMI, WC and WHR according to SC or how much specific SC (e.g., the TE type) is associated with higher risk compared to a reference SC (e.g., the SE type).

Therefore, the purpose of this research was to identify the prevalence of general and abdominal obesity in the Korean population using BMI, WC and WHR according to SC.

## Methods

### Study design and subjects

This was a cross sectional study based on data from the Korean Constitutional Multicenter Study (KCMS) collected between November 2006 and August 2012. The KCMS collected data from 24 Korean medical clinics (KMCs), the details of which have been published in previous studies [19, 24, 25].

We used a sample size of at least 600 subjects of the SE type, which is known to occur in 20% of individuals in Korea population [13]. We also determined a 95% confidence interval and a 4% margin of error.

**Figure 1:**
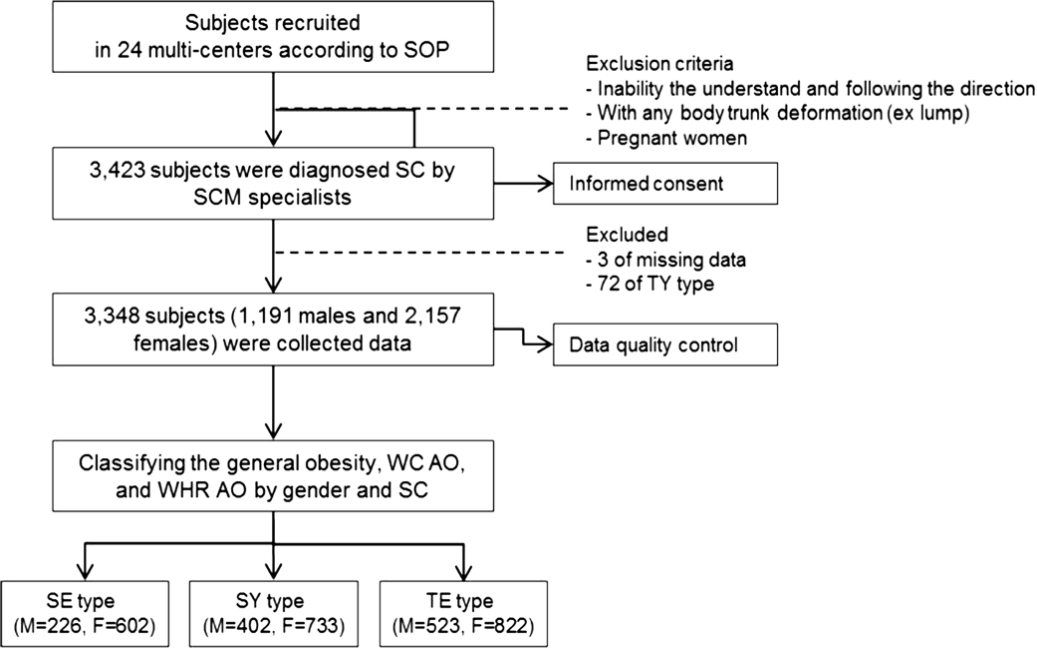
**Flow chart of the study.** SOP, standard operating procedure; SC, Sasang constitution; SCM, Sasang constitutional medicine; TY, Taeyangin; SE, Soeumin; SY, Soyangin; TE, Tae-eumin; BMI, body mass index; WC, waist circumference; WHR, waist-to-hip ratio; AO, abdominal obesity.

The inclusion criteria for the subjects in this study were over 20 years old. The exclusion criteria were as follows: inability to understand and follow the researcher’s indications and inability to stand up or sit down for measurement. Details regarding the researcher’s instructions and the subjects’ measurement postures were described in former reliability study [26]. Subjects with body deformations such as lumps or congenital malformations in the measurement location, as well as pregnant women, were also excluded. Three of them were excluded because of missing data and 72 TY types were also excluded because of their low occurrence in the Korean population. A total of 3,348 subjects (1,191 males and 2,157 females) were included in the final analysis. A flowchart of the study design is shown in Figure 1.

This study was approved by the Korea Institute of Oriental Medicine (KIOM) Institutional Review Board (I-0910/02-001), and signed informed consent was obtained from all subjects. Data collectors were trained rigorously to ensure quality of data acquisition according to the standard operating procedure (SOP) developed by KIOM [26, 27].

### Sasang constitutional diagnosis

The subjects’ SC was diagnosed by SCM specialists at each hospital. To ensure highly accurate SC diagnoses, we applied strict criteria for the SCM specialists and study subjects. SCM specialists had more than 5 years of clinical experience in a medical institution, and the subjects had been administered SC-specific pharmaceuticals before recruitment additionally [19].

### General information and anthropometric measurements

The general information obtained included gender, age, education status (under high school graduates and university graduates), occupation type (white collar and others), and eating habits (amount and speed of eating), which potentially contributed to the SC-obesity association [28, 29].

Anthropometric measurements, including WC, hip circumference, height and weight, which are necessary for the diagnosis of obesity were measured following the SOP [27]. Height and weight were measured in increments of 0.1 cm in height and 0.1 kg in weight. WC at the umbilicus level and HC at the maximal protuberance of the gluteal region were measured in the standing position after the subjects removed the garments from their upper body and stood in an erect posture with their arms folded in front of their chest [26]. BMI was calculated as weight in kilograms divided by height squared in meters after transformation. WHR was also calculated as WC divided by HC.

### Definition of obesity

Obesity was divided into three categories: general obesity, abdominal obesity by waist circumference (WC AO) and abdominal obesity by waist-to-hip ratio (WHR AO). General obesity was defined as BMI ≥25 kg/m^2^, in accordance with the Asia-Pacific criteria of the WHO guidelines [30]. WC AO was defined as WC ≥90 cm in males and ≥85 cm in females, according to the definition by the Korea Society for the Study of Obesity [31]. WHR AO was defined as WHR ≥0.9 in males and ≥0.85 in female according to WHO guidelines [32].

### Statistical analyses

Continuous variables were reported as the mean ± SD, while categorical variables were expressed as percentages. A T test and One-way ANOVA were used to the compare continuous variables (Scheffé's post-hoc analysis) according to gender and SC. A Chi-square test was performed to compare the prevalence of obesity as defined by BMI, WC and WHR according to gender and SC, as well as by 20-year age groups. Logistic regression was used to calculate odds ratios (ORs) for general obesity, WC AO and WHR AO, setting the reference SE type. We conducted all of the analyses using SPSS 17.0 software (SPSS Inc., Chicago, IL). The level of statistical significance was considered to be *p* < .05.

## Results

### Subjects characteristics

Table 1 shows the general and anthropometric characteristics of the subjects by gender and SC. The distribution of the SE, SY and TE types was 22.3%, 33.9% and 43.9% in males and 27.9%, 34% and 38.1% in females, respectively. The mean values of BMI, WC and WHR were significantly different according to SC in both genders, and the TE type was larger than the other types according to Scheffé's post-hoc analysis (*p* < .05).

**Table 1:** **General Characteristics of the subjects**

	SC type				*P*value ^*^
	SE	SY	TE	Total	
**Male**					
N (%)	266 (22.3)	402 (33.9)	523 (43.9)	1191 (100)	0.001
Age (yrs)	46.1 ± 14	51.2 ± 14.2	50.9 ± 14.3	49.9 ± 14.3	<0.001
Height (cm)	169.8 ± 6.1	169 ± 6.2	170.2 ± 6.3	169.7 ± 6.2	0.025
Weight (kg)	63.3 ± 8.3	67.4 ± 8.9	74.8 ± 10.7	69.7 ± 10.7	<0.001
BMI (kg/m2)	21.9 ± 2.4	23.6 ± 2.6	25.8 ± 2.9	24.1 ± 3.0	<0.001
WC (cm)	81.8 ± 7.2	85.9 ± 7.1	92.1 ± 7.8	87.7 ± 8.5	<0.001
WHR	0.9 ± 0.06	0.92 ± 0.05	0.95 ± 0.06	0.93 ± 0.06	<0.001
Education, high school	121 (45.8)	220 (55)	269 (52)	610 (51.7)	0.067
Occupation, white collar	127 (48.8)	138 (35.2)	189 (36.6)	454 (38.8)	0.001
Amount of eating					
too much	15 (5.6)	55 (13.7)	95 (18.2)	165 (13.9)	<0.001
a moderate amount	175 (65.8)	252 (62.7)	317 (60.6)	744 (62.5)	
too little	51 (19.2)	68 (16.9)	58 (11.1)	177 (14.9)	
irregular	25 (9.4)	27 (6.7)	53 (10.1)	105 (8.8)	
Speed of eating					
quickly	136 (51.1)	216 (53.7)	334 (63.9)	686 (57.6)	<0.001
moderately	76 (28.6)	125 (31.1)	134 (25.6)	335 (28.1)	
slowly	54 (20.3)	61 (15.2)	55 (10.5)	170 (14.3)	
**Female**					
N (%)	602 (27.9)	733 (34)	822 (38.1)	2157 (100)	
Age (yrs)	46.9 ± 15	48.3 ± 13.8	51.6 ± 14.9	49.1 ± 14.7	<0.001
Height (cm)	158 ± 5.8	156.8 ± 5.9	157.6 ± 5.8	157.4 ± 5.8	0.001
Weight (kg)	52.6 ± 6.3	55.4 ± 6.8	62.7 ± 8.5	57.4 ± 8.5	<0.001
BMI (kg/m2)	21.1 ± 2.5	22.5 ± 2.7	25.3 ± 3.2	23.1 ± 3.3	<0.001
WC (cm)	77.4 ± 8.2	80.5 ± 8.4	88.3 ± 9.3	82.5 ± 9.8	<0.001
WHR	0.86 ± 0.07	0.88 ± 0.07	0.91 ± 0.07	0.89 ± 0.07	<0.001
Education, high school	331 (55.3)	416 (56.8)	570 (70.2)	1317 (61.5)	0.001
Occupation, white collar	157 (26.2)	201 (27.8)	162 (20.1)	520 (24.4)	<0.001
Amount of eating					
too much	27 (4.5)	47 (6.4)	93 (11.3)	167 (7.7)	<0.001
a moderate amount	361 (60)	438 (59.8)	434 (52.8)	1233 (57.2)	
too little	127 (21.1)	114 (15.6)	133 (16.2)	374 (17.3)	
irregular	87 (14.5)	134 (18.3)	162 (19.7)	383 (17.8)	
Speed of eating					
quickly	209 (34.7)	332 (45.3)	441 (53.6)	982 (45.5)	<0.001
moderately	245 (40.7)	265 (36.2)	260 (31.6)	770 (35.7)	
slowly	148 (24.6)	136 (18.6)	121 (14.7)	405 (18.8)	

Data are the mean ± SD or n (%). ^*^ *p* value was determined by a chi-squared test or one-way ANOVA of among SC by gender. SC, Sasang constitution; SE, Soeumin; SY, Soyangin; TE, Tae-eumin; BMI, body mass index; WC, waist circumference; WHR, waist-to-hip ratio.

### The prevalence of general and abdominal obesity by gender and SC

**Figure 2:**
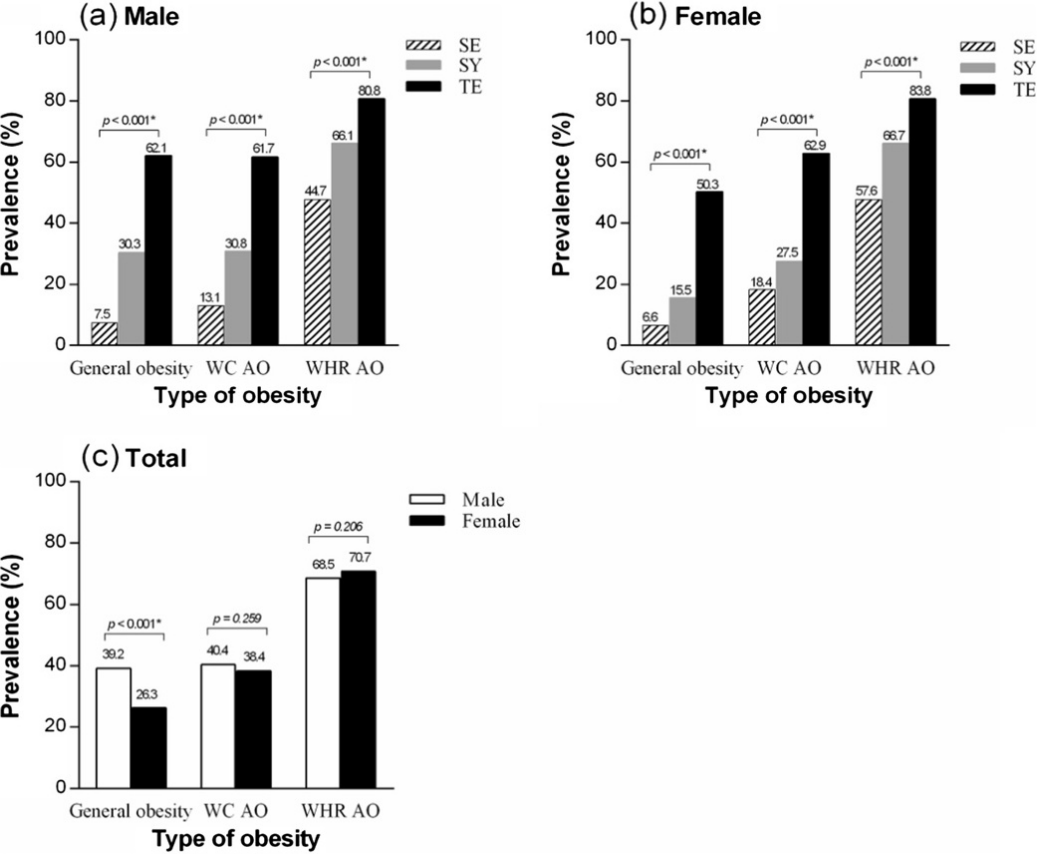
**Prevalence rates of obesity according to gender and SC. (a)** prevalence rates of obesity by SC in male, **(b)** prevalence rates of obesity by SC in female, **(c)** prevalence rates of obesity by gender. ^*^ *p* value < 0.05, *p* value was determined using a chi-square test of prevalence among SC types or gender. SC, Sasang constitution; SE, Soeumin; SY, Soyangin; TE, Tae-eumin; WC, waist circumference; WHR, waist-to-hip ratio; AO, abdominal obesity.

Figure 2 presents the prevalence of general obesity, WC AO and WHR AO according to gender and SC. In males, the prevalence of general obesity, WC and WHR AO was highest in the TE type, with values of 62.1%, 61.7% and 80.8%, respectively (*p* < .001). In females, the prevalence of general obesity, WC and WHR AO was also highest in the TE type at 50.3%, 62.9% and 83.3%, respectively (*p* < .001). The overall prevalence of general obesity was 39.2% and 26.3% for males and females, respectively (*p* < .001), whereas the overall prevalence of WC AO was 40.4% and 38.4% (*p* = .259), and that of WHR AO was 68.5% and 70.7% for males and females (*p* = .206), respectively.

### The prevalence of general and abdominal obesity by age, gender and SC

**Figure 3:**
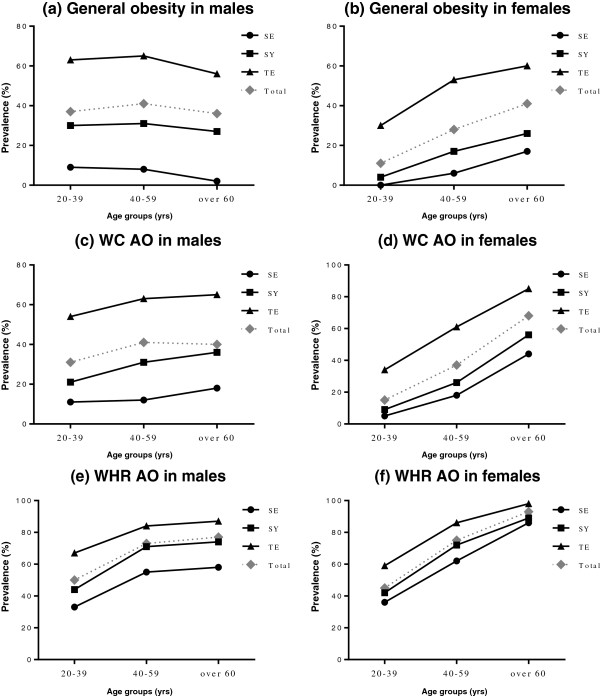
**Prevalence rates of obesity by age according to gender and SC (a) and (b): general obesity, (c) and (d): WC AO, (e) and (f): WHR AO in male and female, respectively.** SE, Soeumin; SY, Soyangin; TE, Tae-eumin; WC, waist circumference; WHR, waist-to-hip ratio; AO, abdominal obesity.

Figure 3 shows the prevalence rates for general obesity, WC AO and WHR AO by age group according to gender and SC; the prevalence rates were significantly different in each age group among SC types (all *p* < .001). Most prevalence rates for general obesity, WC AO and WHR AO increased following age increases in each gender and SC. The TE type had the highest prevalence, and the SE type had the lowest prevalence of general and abdominal obesity among all SC types for all age groups and both genders.

### ORs for general and abdominal obesity according to gender and SC

Table 2 shows a sequentially developed logistic regression model of obesity according to gender and SC. The results revealed that general obesity, WC AO and WHR AO were inversely associated with SC type. The TE and SY types were significantly associated with an increased risk of general obesity, WC AO and WHR AO, compared with the SE type as reference in both males and females. In addition, after controlling for age, social status and eating habits, the ORs were similar to those of the crude model according to gender and SC. Males showed significantly increased ORs with respect to general obesity compared with females in crude and adjusted confounders.

**Table 2:** **Odds ratios (95% CI) of obesity classified by BMI, WC and WHR according to gender and SC**

		SC type			Total ^*^
		SE	SY	TE	
Male					
General obesity	Crude	1.0	5.4 (3.2-8.9)	20.2 (12.4-32.9)	1.8 (1.5-2.1)
	Adjusted	1.0	5.2 (3.1-8.7)	18.1 (11.0-30.0)	1.8 (1.5-2.2)
WC AO	Crude	1.0	2.9 (1.9-4.5)	10.7 (7.2-15.9)	1.1 (0.9-1.2)
	Adjusted	1.0	2.4 (1.5-3.7)	8.6 (5.7-12.9)	1.1 (0.9-1.3)
WHR AO	Crude	1.0	2.1 (1.6-2.9)	4.6 (3.3-6.4)	0.9 (0.7-1.1)
	Adjusted	1.0	1.73 (1.2-2.4)	3.9 (2.7-5.6)	0.8 (0.7-1.0)
Female					
General obesity	Crude	1.0	2.6 (1.8-3.8)	14.3 (10.1-20.2)	-
	Adjusted	1.0	2.3 (1.5-3.4)	11.6 (8.1-16.7)	-
WC AO	Crude	1.0	1.7 (1.3-2.2)	7.5 (5.8-9.6)	-
	Adjusted	1.0	1.6 (1.2-2.1)	7.0 (5.3-9.3)	-
WHR AO	Crude	1.0	1.4 (1.2-1.8)	3.8 (2.9-4.9)	-
	Adjusted	1.0	1.3 (1.1-1.7)	3.1 (2.3-4.1)	-

Adjusted for age, education, occupation, and eating habits such as amount and speed.

^*^ORs (95% CI) for obesity in males compared with females as reference.

SC, Sasang constitution; SE, Soeumin; SY, Soyangin; TE, Tae-eumin; WC, waist circumference; WHR, waist-to-hip ratio; AO, abdominal obesity; CI, confidence interval.

## Discussion

The aim of the present study was not only to reveal a clinical association between Sasang constitution and the prevalence of general and abdominal obesity, but also to determine whether a SC (TE type) is associated with greater risk compared to reference SC (SE type).

This study revealed some different prevalence trends in general obesity between males and females. The prevalence of general obesity as determined by BMI was higher in males than in females, whereas the prevalence of AO by WC and WHR was similar in males and females. Furthermore, the prevalence of general obesity was similar to that of WC AO in males, although it was relatively lower than that of WC AO in females. This reconfirmed that obesity type could differ according to gender. This finding corresponds well with reports from the Korea National Health and Nutrition Examination Surveys [33]. These findings are indirect evidence that subjects recruited from 24 KMC for this study may be representative of the Korean population. Overall, the prevalence of WHR AO was much higher than that of WC AO. Previous studies have reported that WHR AO in older females was relatively high and increased with age [34, 35]. Considering this information, we might assume that the old average age of the subjects may be associated with higher WHR AO.

Our results also clarify the assumption that the TE type might be much more associated with overweight and obese than the SE and SY types, which has been implied in previous published studies [16–19, 23]. This study also demonstrated that the prevalence of obesity as indicated by BMI, as well as WC, was higher in the TE type than in the other types. Furthermore, a new finding is that WHR in the TE type is much larger than in the other SC types, resulting in higher WHR AO prevalence.

Because age is associated with obesity [36] and was significantly different according to SC in this study, the data were analyzed according to stratified age groups for each gender. The prevalence of WC AO continuously increased as age increased in both genders [33, 37, 38]. While females exhibited an increasing trend in the prevalence of general and abdominal obesity with age, the prevalence of general obesity in males peaked in middle-aged groups and decreased in older groups. This finding indicates that middle-aged males are more prone to general obesity than older males, whereas older females are much more susceptible to general and abdominal obesity than middle-aged females. In this study, older females tended to have a higher prevalence of both general and central obesity. We might cautiously suggest that this difference is the result of the menopause status of female participants as the age because previous studies have associated menopause status with increased general and abdominal obesity due to the accumulation of body fat distribution in aging female [39–41].

Among the SC types, the TE type had the highest prevalence rates of general and abdominal obesity in all age groups. The age-specific obesity prevalence trends according to SC type were similar to those of metabolic syndrome [19]. This relatively higher prevalence of general and abdominal obesity in the TE type and the relatively lower prevalence in the SE type in all age groups were similar in males and females; these prevalence rates appeared to be related to the characteristics of individual SC types.

This study suggests the TE type is associated with higher rates of general obesity (OR = 20.2 for males, OR = 14.3 for females), WC AO (OR = 10.7 for males, OR = 7.5 for females), and WHR AO (OR = 4.6 for males, OR = 3.8 for females) than are the SE and SY types in both males and females. The findings indicate that the TE type may be most susceptible to all types of obesity, including general obesity, WC AO and WHR AO, ranked from highest to lowest prevalence.

This study is meaningful by suggesting that SC may be associated with various obesities. In particular, the TE type was the highest risk factor for obesity, which many previous studies did not show, even though they hinted that TE types might gain much more weight than other types. Second, we applied WHR AO as an AO index following the WHO guidelines and proposed trends according to age increases.

However, this study had some limitations. This study has a cross-sectional design, which cannot demonstrate causal effects and time trends. Therefore, the evidence may be slightly weak. We did not exclude body shape influences from the SC diagnoses conducted by specialists. As such, the SC diagnoses by the SCM specialists may have been dependent on or at least affected by the patient’s body shape.

We tried to adjust for confounders such as social status and eating habits, but we did not control for other environmental factors that can influence obesity, such as lifestyle, exercise habits, smoking, physical activity and psychosocial factors, all of which are known to affect body shape. Finally, it is controversial that a pure SC effect, excluding the effect of weight gain, may exist or influence specific diseases that have already been known to result from obesity. We think further research is warranted to identify a pure SC effect by controlling for environmental factors and BMI.

## Conclusion

This study showed that the prevalence of obesity varies according to SC. In particular, the TE type was highly associated with increased ORs of general and abdominal obesity in both genders and in all age groups.
